# Evidence for treating malaria with artemisinin-based combination therapy in the first trimester of pregnancy

**DOI:** 10.1016/S1473-3099(20)30131-6

**Published:** 2020-04-29

**Authors:** Julie R Gutman, R Matthew Chico

**Affiliations:** Malaria Branch, Division of Parasitic Diseases and Malaria, Center for Global Health, US Centers for Diseases Control and Prevention, Atlanta, GA 30329, USA (JRG); Department of Disease Control, Faculty of Infectious and Tropical Disease, London School of Hygiene & Tropical Medicine, London, UK (RMC)

Treatment of malaria during pregnancy requires balancing the need for radical cure while avoiding teratogenic exposure. In *The Lancet Infectious Diseases*, Makoto Saito and colleagues report the results of a systematic review and meta-analysis that used individual patient data on antimalarial efficacy and tolerability in pregnancy.^1^ This impressive undertaking comprises nearly all published evidence and clearly highlights the superiority of artemisinin-based combination therapy (ACT) over quinine monotherapy, building on previous studies that focused on ACT safety in the second and third trimesters.^2^

Quinine has been the only treatment for malaria in much of the world over the past four centuries, with the first reported use for a microscopically confirmed diagnosis of malaria during pregnancy in 1908.^3^ WHO recommends quinine for the treatment of malaria in the first trimester, ideally in combination with clindamycin. First-trimester use of ACTs is only advised when quinine is not available.^4^ However, because clindamycin is rarely available at the point of care in malaria-endemic countries, the vast majority of pregnant women diagnosed with first-trimester malaria are given quinine monotherapy, or an ACT if pregnancy is not recognised.^5,6^

Saito and colleagues found that quinine plus clindamycin had a similar PCR-corrected efficacy on day 28 compared to artemether-lumefantrine (AL; 99·9% *vs* 96·9%, respectively; adjusted hazard ratio 0·48 [95% CI 0·04–5·24]), whereas quinine monotherapy had an efficacy of only 87·7% (6·11, 2·57–14·57) compared to AL. The substantially lower efficacy of quinine monotherapy versus AL is further evidence to support the change to WHO’s treatment guidelines recommended by the Malaria Policy Action Committee in 2016.^7^

Although only 33 of 4968 total episodes in the meta-analysis were in their first trimester, there is no evidence to suggest that the relative efficacy of antimalarials in the first trimester would differ from treatment later in pregnancy. Moreover, the higher efficacy of artemisinin over quinine is more apparent in severe disease for which parenteral artemisinin is already the preferred option.^4^

It is difficult to draw conclusions regarding safety given the very small number of first-trimester pregnancies in this study. However, the safety of ACT in pregnancy is supported by data for 30618 pregnancies (947 exposed to quinine and 717 exposed to ACTs in the first trimester) that showed that the risk of miscarriage and stillbirth are similar between ACTs and quinine. Compared with quinine, the risk of ACTs on miscarriage were 0·73 (95% CI 0·44–1·21) and the risk of ACT on stillbirth were 0·29 (0·08–1·02).^8^ Neither ACTs nor quinine were associated with an increased risk of stillbirth compared with no antimalarial treatment in the first trimester. Quinine administered in the first trimester was associated with increased risk of miscarriage compared with no antimalarial treatment, although ACTs were not. The risks of congenital malformations from quinine and ACTs were similar. These numbers are only sufficient to exclude a greater than approximately two-fold increase in the risk of miscarriage and stillbirth, underscoring the need for more data.^8^ However, it has taken two decades to collect the evidence to date, and it is unlikely that more data will be available soon. There is also the issue of poor tolerability associated with quinine, whereby a very high proportion of patients reported symptoms of cinchonism, including tinnitus, by day 7 of therapy.^9^ These adverse effects might be so bothersome that therapy is discontinued prematurely, further reducing its efficacy. Effective treatment clearly reduces the risk of stillbirth caused by malaria infection.^10^ Therefore, even if it was ensured that all first-trimester women with parasitaemia receive quinine plus clindamycin for 7 days, these patients might not be optimally served.

Today, there are insufficient safety data for the use of ACTs in first trimester to argue for their use in first trimester for scenarios in which malaria testing is not done before dosing (eg, mass drug administration) or for scenarios in which the drug is provided prophylactically (eg, intermittent preventive treatment in pregnancy). However, considering the evidence base of efficacy, adherence, and tolerability, the risk-benefit analysis for treatment favours the use of ACTs for confirmed malaria in the first trimester of pregnancy.^7^

## References

[1] Saito M, Mansoor R, Kennnon K, Efficacy and tolerability of artemisinin-based and quinine-based treatments for uncomplicated falciparum malaria in pregnancy: a systematic review and individual patient data meta-analysis. Lancet Infect Dis 2020; published online April 29. 10.1016/S1473-3099(20)30064-5. PMC7391007 32530424

[2] Kovacs SD, van Eijk AM, Sevene E, The safety of artemisinin derivatives for the treatment of malaria in the 2nd or 3rd trimester of pregnancy: a systematic review and meta-analysis. PLoS One 2016;11: e0164963. 27824884 10.1371/journal.pone.0164963 PMC5100961

[3] Maxwell JP. The use of quinine during pregnancy, labour and the puerperium. J Trop Med Hyg 1908; 11: 191–94.

[4] WHO. Guidelines for the treatment of malaria, 3rd edn. Geneva: World Health Organization, 2015. 26020088

[5] Hill J, D’Mello-Guyett L, Hoyt J, van Eijk AM, ter Kuile FO, Webster J. Women’s access and provider practices for the case management of malaria during pregnancy: a systematic review and meta-analysis. PLoS Med 2014; 11: e1001688. 25093720 10.1371/journal.pmed.1001688 PMC4122360

[6] Riley C, Dellicour S, Ouma P, Knowledge and adherence to the national guidelines for malaria case management in pregnancy among healthcare providers and drug outlet dispensers in rural, western Kenya. PLoS One 2016; 11: e0145616. 26789638 10.1371/journal.pone.0145616 PMC4720358

[7] WHO malaria policy advisory committee and secretariat. Malaria policy advisory committee to the WHO: conclusions and recommendations of eighth biannual meeting (September 2015). Malar J 2016; 15: 117. 26911803 10.1186/s12936-016-1169-x PMC4766637

[8] Dellicour S, Sevene E, McGready R, First-trimester artemisinin derivatives and quinine treatments and the risk of adverse pregnancy outcomes in Africa and Asia: a meta-analysis of observational studies. PLoS Med 2017; 14: e1002290. 28463996 10.1371/journal.pmed.1002290 PMC5412992

[9] White NJ, Looareesuwan S, Warrell DA, Warrell MJ, Bunnag D, Harinasuta T. Quinine pharmacokinetics and toxicity in cerebral and uncomplicated Falciparum malaria. Am J Med 1982; 73: 564–72. 6751085 10.1016/0002-9343(82)90337-0

[10] Moore KA, Simpson JA, Scoullar MJL, McGready R, Fowkes FJI. Quantification of the association between malaria in pregnancy and stillbirth: a systematic review and meta-analysis. Lancet Glob Health 2017; 5: e1101–12. 28967610 10.1016/S2214-109X(17)30340-6

